# Regulation of the Nur77-P2X7r Signaling Pathway by Nodakenin: A Potential Protective Function against Alcoholic Liver Disease

**DOI:** 10.3390/molecules29051078

**Published:** 2024-02-29

**Authors:** Jian Song, Bo-Feng Qin, Jin-Jin Zhang, Qi-Yuan Feng, Guan-Cheng Liu, Gui-Yun Zhao, Hai-Ming Sun

**Affiliations:** 1 College of Pharmacy, Beihua University, Jilin 132013, China; songjianybu@beihua.edu.cn (J.S.); qbf1380@163.com (B.-F.Q.); zjj18755366801@163.com (J.-J.Z.); yy18643409789@163.com (Q.-Y.F.); lgc531835978@163.com (G.-C.L.); 2 College of Science, Traditional Chinese Medicine Biotechnology Innovation Center in Jilin Province, Beihua University, Jilin 132013, China

**Keywords:** Nodakenin, alcoholic liver disease, inflammatory, Nur77, P2X7r

## Abstract

Alcoholic liver disease (ALD) is the main factor that induces liver-related death worldwide and represents a common chronic hepatopathy resulting from binge or chronic alcohol consumption. This work focused on revealing the role and molecular mechanism of nodakenin (NK) in ALD associated with hepatic inflammation and lipid metabolism through the regulation of Nur77-P2X7r signaling. In this study, an ALD model was constructed through chronic feeding of Lieber–DeCarli control solution with or without NK treatment. Ethanol (EtOH) or NK was administered to AML-12 cells, after which Nur77 was silenced. HepG2 cells were exposed to ethanol (EtOH) and subsequently treated with recombinant Nur77 (rNur77). Mouse peritoneal macrophages (MPMs) were treated with lipopolysaccharide/adenosine triphosphate (LPS/ATP) and NK, resulting in the generation of conditioned media. In vivo, histopathological alterations were markedly alleviated by NK, accompanied by reductions in serum triglyceride (TG), aspartate aminotransferase (AST), and alanine aminotransferase (ALT) levels and the modulation of Lipin-1, SREBP1, and Nur77 levels in comparison to the EtOH-exposed group (*p* < 0.001). Additionally, NK reduced the production of P2X7r and NLRP3. NK markedly upregulated Nur77, inhibited P2X7r and Lipin-1, and promoted the function of Cytosporone B, a Nur77 agonist (*p* < 0.001). Moreover, Nur77 deficiency weakened the regulatory effect of NK on P2X7r and Lipin-1 inhibition (*p* < 0.001). In NK-exposed MPMs, cleaved caspase-1 and mature IL-1β expression decreased following LPS/ATP treatment (*p* < 0.001). NK also decreased inflammatory-factor production in primary hepatocytes stimulated with MPM supernatant. NK ameliorated ETOH-induced ALD through a reduction in inflammation and lipogenesis factors, which was likely related to Nur77 activation. Hence, NK is a potential therapeutic approach to ALD.

## 1. Introduction

Alcoholic liver disease (ALD) is a major cause of acute and chronic liver injury and can include simple steatosis, steatohepatitis, advanced fibrosis, cirrhosis, and even hepatocellular carcinoma [1,2]. ALD can be caused by byproducts or metabolites produced during alcohol metabolism and is a major factor that induces chronic liver disease [3]. Alcohol metabolism promotes the transition from alcoholic fatty liver disease to alcoholic steatohepatitis (ASH), which is characterized by steatosis, ballooning hepatocytes, and macrophage infiltration. Kupffer cells are also activated to release chemokines and ROS, thereby recruiting blood-derived monocytes and bone-marrow-derived neutrophils to the liver in patients with ASH [4,5,6]. Along with the ubiquitous research and development of ALD, there has been sustained interest in the complex mechanisms linking hepatocyte injury to inflammation.

Nur77 (NR4A1), an orphan member of the nuclear receptor superfamily, has a critical effect on various cellular processes, such as survival, apoptosis, autophagy, inflammation, and immunity, in response to diverse stimuli [7]. Nur77 can be distributed within various metabolism-related tissues, including adipose tissue, liver tissue, skeletal muscle, and pancreatic tissue [8]. As a transcription factor, Nur77 can exert its hepatoprotective effects by regulating glucose and lipid homeostasis. Research has shown that Nur77 participates in regulating obesity and related metabolic diseases, such as fatty liver disease and hyperlipidemia [9]. Nur77 upregulation promotes liver glucose generation, increases blood glucose content, and activates genes related to gluconeogenesis [10,11]. However, Nur77 is also vital for the inflammatory response. Nur77 is considered an important factor regulating hepatic inflammation and fibrogenesis, and suppressing its expression is closely related to the activation of hepatic stellate cells (HSCs) [12]. Elevated extracellular nucleotide levels are a marker of inflammatory conditions; specifically, ATP can promote the inflammatory response through purinergic receptors [13]. As the ionic receptor of the purinergic P2 receptor family, the P2X7 receptor (P2X7r) has an effect on inflammatory reactions in various diseases [14]. ATP is suggested to intensify inflammation by upregulating P2X7r while promoting cytokine production, thereby aggravating liver injury. In alcoholic liver patients, P2X7r can respond to metabolic and ethanol stimuli early [15,16]. P2X7r can also act on the nucleotide-binding oligomerization domain-like receptor protein 3 (NLRP3) inflammasome while promoting liver inflammation by recruiting and activating the NLRP3 inflammasome to mediate IL-1β maturation [17]. Given that Nur77 is important for liver disease progression, inflammation, and lipid accumulation, the molecular mechanism regulating the Nur77-P2X7r pathway deserves further exploration.

Nodakenin (NK), a furanocoumarin glycoside initially obtained from Angelicae gigas roots, has been shown to have anti-inflammatory, antioxidant, and antihyperglycemic effects [18,19,20,21]. Recent research has shown that NK is a potential hepatoprotective agent. NK pretreatment inhibited apoptosis-related factors, oxidative stress mediators, and inflammatory response factors in LPS-treated mouse livers. NK administration also alleviated high-fat-diet-induced obesity via the inhibition of oxidative-stress-mediated adipogenesis [22]. Although it has been reported that NK alleviates chronic liver injury, the exact mechanism through which NK affects inflammation in ALD is still unclear. Here, we explored the effect of the Nur77-P2X7r axis on ALD pathogenesis. Regulating the Nur77-P2X7r axis may constitute a novel molecular mechanism for NK-mediated treatment of ALD.

## 2. Result

### 2.1. NK Regulates Lipid Deposition and Inflammatory Response in EtOH-Stimulated AML-12 Cells

**Figure 1:**
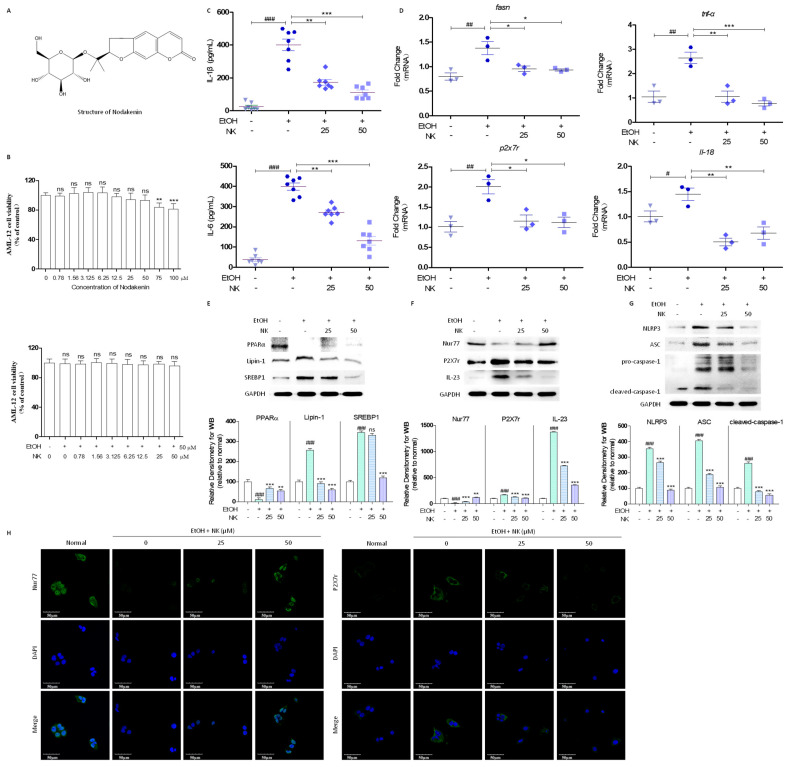
NK Regulates Lipid Deposition and Inflammatory Response in EtOH-Stimulated AML-12 Cells. (**A**) Chemical structure of Nodakenin. (**B**) MTT assay measuring cell viability of AML-12 with NK treatment. (**C**) The IL-1β and IL-6 protein levels released from AML-12 cells into culture medium were determined by an ELISA assay. (**D**) Real-time PCR was used to determine the mRNA expression of *fasn*, *p2x7r*, *tnf-α,* and *Il-18*. (**E**) Representative western blotting analysis for expression of PPARα, Lipin-1, and SREBP1. (**F**) Representative western blotting analysis for expression of Nur77, P2X7r, and IL-23. (**G**) Representative western blotting analysis for expression of NLRP3, ASC, and caspase-1. GAPDH was used as the loading control. (**H**) Immunofluorescence staining of Nur77 and P2X7r presented at 600× magnification. ^#^ *p* < 0.05, ^##^ *p* < 0.01, ^###^ *p* < 0.001 vs. normal group; * *p* < 0.05, ** *p* < 0.01, *** *p* < 0.001 vs. TGF-β group.

This study analyzed how NK affects AML-12 cell viability through MTT assays. NK (0–100 µM) was added to AML-12 cells for a 24 h period. The results showed that NK (0–50 µM) did not affect AML-12 cell viability (Figure 1B). Additionally, we detected the effects of EtOH (50 mM) and NK (0–50 μM) on the cell viability of AML-12 cells for 24 h (Figure 1B). EtOH (50 mM) and NK (0–50 μM) exposure for 24 h did not significantly decrease cell viability compared with the negative control. Lipin1 is a protein generated by the LPIN1 gene and can interact with other nuclear receptors to regulate lipid metabolism; such receptors include SREBP1 and PPARα/γ. The activation of NLRP3 facilitates the recruitment of ASC, which recruits procaspase-1, resulting in its cleavage and activation and thereby inducing the secretion of inflammatory cytokines. Ethanol stimulation increased the release of IL-1β and IL-6 by AML-12 cells. However, treatment with NK had the opposite effect (Figure 1C). In EtOH-exposed AML-12 cells, the *fasn*, *p2x7r*, *tnf-α*, and *Il-18* mRNA levels were significantly increased, while NK treatment effectively downregulated these mRNA levels relative to those in EtOH-treated cells (Figure 1D). Compared to negative control cells, AML-12 cells significantly expressed Lipin-1 and SREBP1 but had significantly reduced PPARα protein levels after EtOH treatment. NK administration markedly decreased Lipin1 and SREBP1 levels but increased PPARα levels relative to those in the EtOH stimulation group (Figure 1E). Compared with that in the normal group, the expression of Nur77 in the EtOH-treated group was reduced. Additionally, NK treatment significantly elevated the expression of Nur77 (Figure 1F). Immunocytochemical staining revealed that NK significantly increased the number of Nur77-positive cells compared to that in the EtOH group (Figure 1H). Upon EtOH treatment, AML-12 cells exhibited elevated protein levels of P2X7r, the NLRP3 inflammasome, and IL-23, as supported by the immunocytochemistry analysis results, which revealed an increase in the P2X7r-positive area in EtOH-treated cells. The administration of NK considerably decreased the P2X7r, NLRP3, caspase-1, ASC, and IL-23 protein levels (Figure 1F–H). Therefore, NK is sufficient to inhibit ALD through the suppression of hepatocyte lipid deposition and inflammatory reactions.

### 2.2. NK Ameliorated ALD via Enhanced Nur77-Mediated P2X7r Signaling and Lipid Accumulation

**Figure 2:**
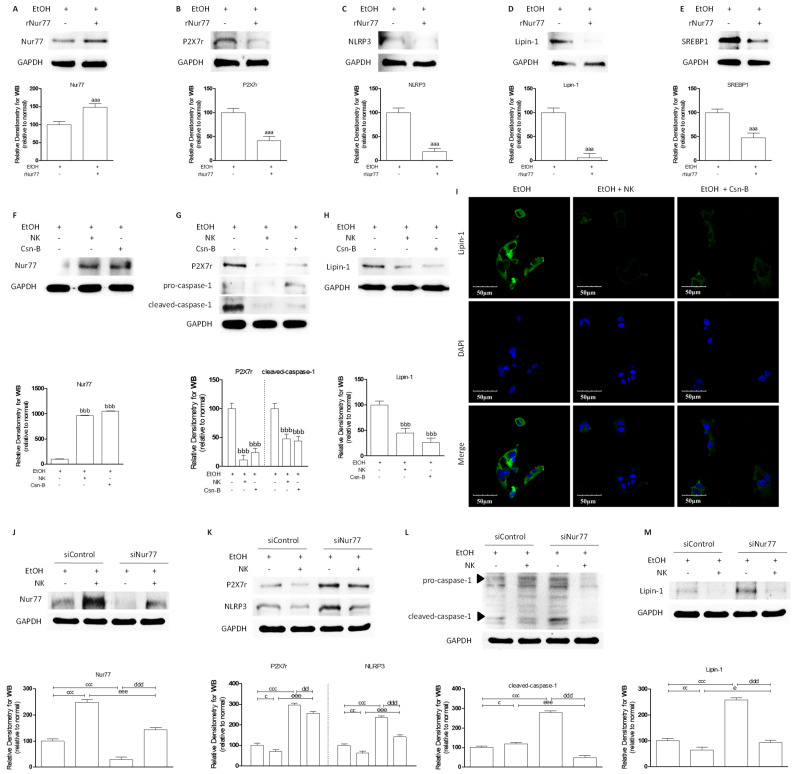
NK Ameliorated ALD via Enhanced Nur77-Mediated P2X7r Signaling and Lipid Accumulation. Representative western blotting analysis for expression of (**A**) Nur77, (**B**) P2X7r, (**C**) NLRP3, (**D**) Lipin-1, and (**E**) SREBP1. GAPDH was used as the loading control. ^aaa^ *p* < 0.001 vs. EtOH group. Representative western blotting analysis for expression of (**F**) Nur77, (**G**) P2X7r, caspase-1, and (**H**) Lipin-1. GAPDH was used as the loading control. ^bbb^ *p* < 0.001 vs. EtOH group. (**I**) Immunofluorescence staining of Lipin-1 presented at 600× magnification. Representative western blotting analysis for expression of (**J**) Nur77, (**K**) P2X7r, NLRP3, (**L**) caspase-1, and (**M**) Lipin-1. GAPDH was used as the loading control. ^c^ *p* < 0.05, ^cc^ *p* < 0.01, ^ccc^ *p* < 0.001 siControl vs. siControl-NK, siRNA (Nur77)-NK groups; ^dd^ *p* < 0.01, ^ddd^ *p* < 0.001 siRNA (Nur77) vs. siRNA (Nur77)-NK group; ^e^ *p* < 0.05, ^eee^ *p* < 0.001 siControl-NK vs. siRNA (Nur77)-NK group.

EtOH-induced hepatocyte injury serves as an indicator of ALD. Nur77 can potentially impede ALD through the inhibition of proinflammatory-mediator and lipid-mediator synthesis. Recombinant human Nur77 protein (rNur77) was administered to HepG2 cells. In addition, HepG2 cells were stimulated with EtOH, which resulted in low Nur77 expression and high P2X7r, NLRP3, Lipin-1, and SREBP1 expression. As predicted, rNur77 administration promoted Nur77 protein expression but decreased P2X7r, NLRP3, Lipin-1, and SREBP1 protein expression in HepG2 cells stimulated with EtOH (Figure 2A–E). Nur77 inhibited the P2X7r signaling pathway and lipid accumulation in EtOH-stimulated HepG2 cells. However, whether NK depended on activated Nur77 to improve ALD needed to be determined. Cytosporone B (Csn-B, a Nur77 agonist) was used for the following experiments. NK apparently upregulated Nur77 protein expression relative to that in the EtOH group (Figure 2G). Moreover, NK dramatically suppressed P2X7r, caspase-1, and Lipin-1 protein expression levels compared with those in the EtOH group, but these changes were not significantly different from those in the Csn-B group (Figure 2H–J). Immunofluorescence staining revealed a noticeable reduction in Lipin-1 expression in the groups treated with NK or Csn-B compared to the EtOH group (Figure 2F). Additionally, NK inhibited Lipin-1 expression that was induced by EtOH (Figure 2F). These results suggest that, similar to Csn-B, NK can potentially function as a Nur77 agonist. To further investigate the anti-ALD effects of NK through Nur77-mediated P2X7r signaling and lipid accumulation, AML-12 cells were subjected to Nur77 gene knockdown and subsequently treated with either EtOH or NK or left untreated. The results showed that siRNA-mediated knockdown of Nur77 markedly decreased Nur77 protein levels (Figure 2K). However, NK could activate the protein expression of Nur77. The EtOH-mediated increases in P2X7r, NLRP3, cleaved caspase-1, and Lipin-1 were strengthened by the Nur77 siRNA. NK could further attenuate the expression of P2X7r, NLRP3, cleaved caspase-1, and Lipin-1 (Figure 2K–M). These results suggest that activated Nur77 is a possible anti-ALD therapeutic target of NK.

### 2.3. NK Attenuates the Inflammatory Response to Macrophage-to-Hepatocyte Communication

**Figure 3:**
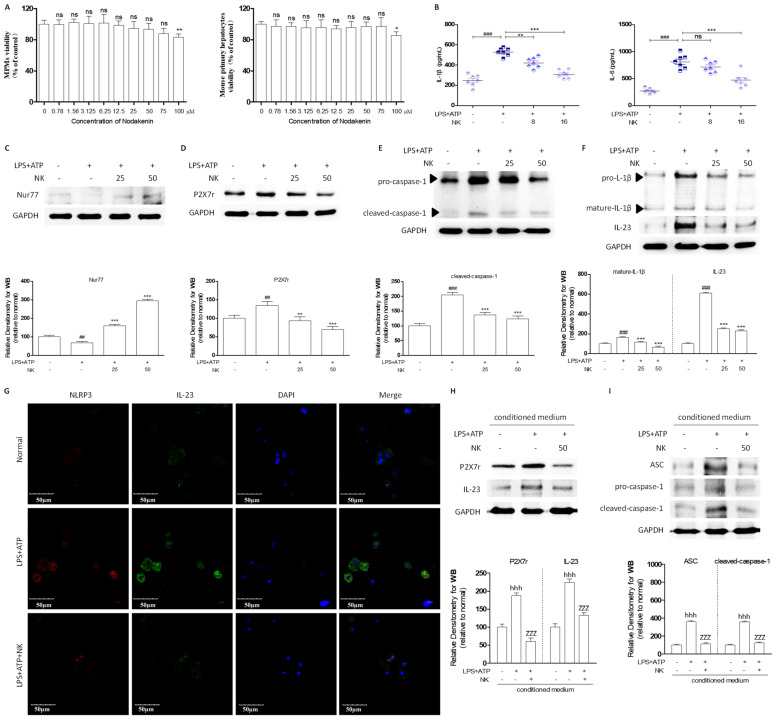
NK Attenuates the Inflammatory Response to Macrophage-to-Hepatocyte Communication. (**A**) MTT assay measuring cell viability of MPMs and mouse primary hepatocytes with NK treatment. * *p* < 0.05 vs. negative control; (**B**) The IL-1β and IL-6 protein released from MPMs into culture medium was measured by an ELISA assay. Representative western blotting analysis for expression of (**C**) Nur77, (**D**) P2X7r, (**E**) caspase-1, (**F**) IL-1β and IL-23. GAPDH was used as the loading control. ^##^ *p* < 0.01, ^###^ *p* < 0.001 vs. normal group; ** *p* < 0.01, *** *p* < 0.001 vs. LPS+ATP group. (**G**) Immunofluorescence staining of NLRP3 and IL-23 presented at 600× magnification. Representative western blotting analysis for expression of (**H**) P2X7r, IL-23, (**I**) ASC, and caspase-1 in primary hepatocytes. Densitometric values were normalized to GAPDH. ^hhh^ *p* < 0.001 vs. normal group; ^zzz^ *p* < 0.001 vs. LPS+ATP group; ns, not significant.

This study used mouse peritoneal macrophages and primary hepatocytes to further validate the ability of NK to ameliorate alcoholic hepatitis. The viability of MPMs and primary hepatocytes cultured with NK (0–100 µM) for a 24 h period was evaluated through an MTT assay. Compared with the negative control, NK (75–100 µM) apparently decreased the number of MPMs and the viability of hepatocytes (Figure 3A). The combination of LPS and ATP significantly increased the release of IL-6 and IL-1β by peritoneal macrophages. However, treatment with NK decreased their secretion (Figure 3B). Compared with that in the normal group, the expression of Nur77 in the LPS/ATP administration group was reduced. However, NK treatment significantly elevated the expression of Nur77 (Figure 3C). As shown in Figure 3D–F, costimulation with LPS and ATP resulted in enhanced expression of P2X7r in MPMs, as well as the production and secretion of the proinflammatory cytokines cleaved caspase-1, IL-1β, and IL-23. NK has been shown to exert a substantial inhibitory effect on the expression of these genes (Figure 3D–F).

To examine the impact of activated macrophages on ALD, conditioned media derived from peritoneal macrophages following stimulation with LPS/ATP were used to treat primary mouse hepatocytes. Notably, the application of conditioned media led to a marked increase in inflammatory-factor levels in primary mouse hepatocytes compared to those in the normal group (Figure 3H–J). Hence, activated macrophages enhance the inflammatory response of hepatocytes. Hepatocytes were exposed to the conditioned media of macrophages that were activated by LPS/ATP, with or without the addition of NK, for the detection of inflammatory factors within hepatocytes. All the NK treatments markedly reduced the P2X7r, ASC, caspase-1, and IL-23 increases in the conditioned media, as supported by the immunocytochemistry analysis results. In addition, NK decreased the number of NLRP3- and IL-23-positive cells in the LPS/ATP-treated primary mouse hepatocytes (Figure 3G–J). According to the above findings, activated macrophages promote the hepatocellular inflammatory response, and the inflammatory cascade subsides in response to NK interference.

### 2.4. NK Improves Liver Injury in ALD Mice

**Figure 4:**
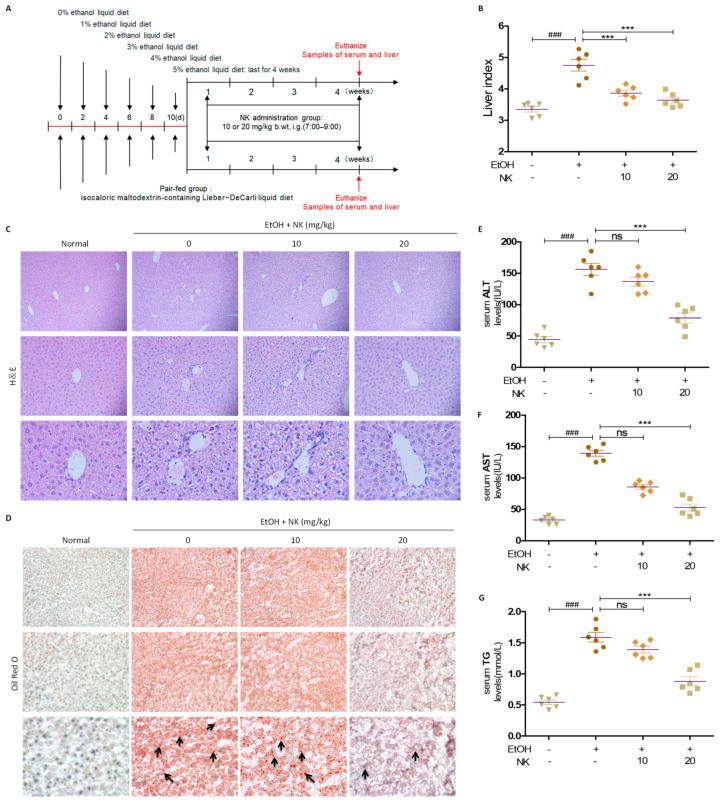
NK Improves Liver Injury in ALD Mice. (**A**) Chronic ethanol-feeding model. (**B**) Liver index of mice. (**C**) H&E staining presented at 100×, 200×, and 400× magnification. (**D**) Oil Red O staining presented at 100×, 200×, and 400× magnification. Black arrows indicate hepatic lipid droplets. Serum (**E**) ALT, (**F**) AST, and (**G**) TG levels. ^###^ *p* < 0.001 vs. normal group; *** *p* < 0.001 vs. EtOH group; ns, not significant.

As shown in Figure 4B, compared with those receiving a normal diet or DG, C57BL/6 mice receiving an alcohol-containing Lieber–DeCarli liquid diet for 4 weeks had an increased liver index. According to Oil Red O and H&E results, obvious lipid droplets could be observed within the livers of chronically ethanol-fed mice, while NK dose-dependently weakened the lipid deposition and liver injury resulting from ethanol consumption (Figure 2B–D). As expected, NK dose-dependently decreased the hepatic histopathology alterations and decreased the excessive liver indices resulting from alcohol consumption (Figure 4B–D). The serum AST, ALT, and TG levels in the EtOH group were apparently elevated, with an obvious recovery trend after NK administration (Figure 4E–G).

### 2.5. NK Regulates Lipid Accumulation in ALD Mice

**Figure 5:**
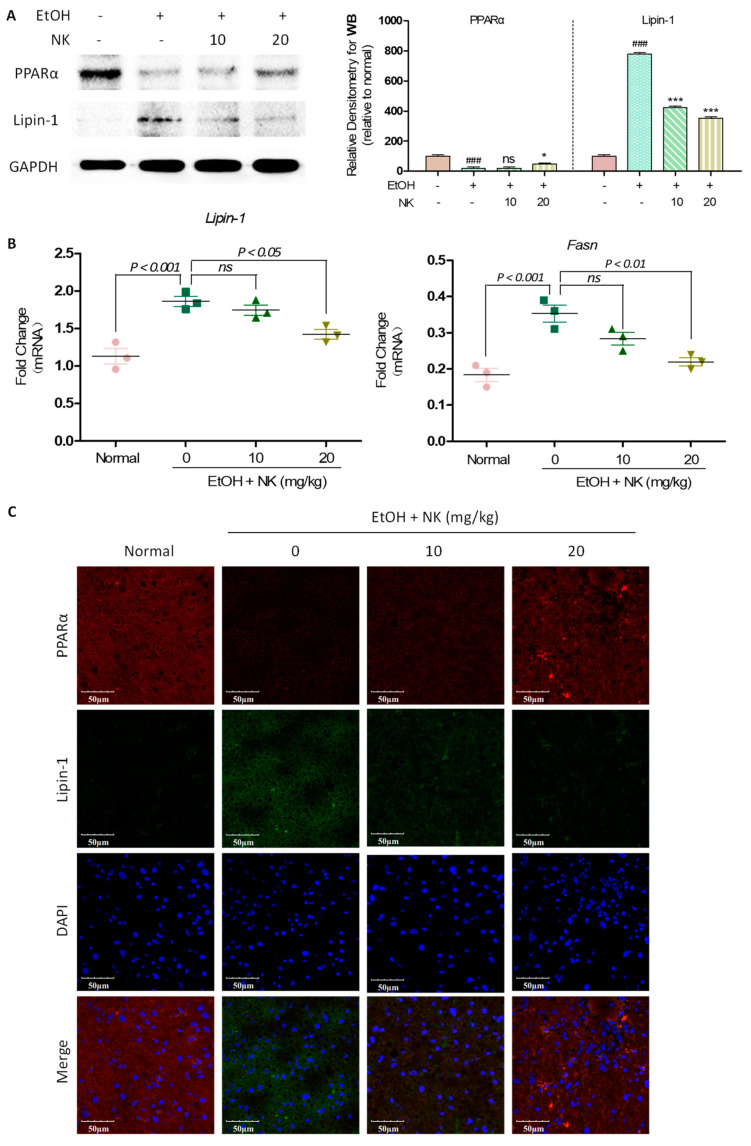
NK Regulates Lipid Accumulation in ALD Mice. (**A**) Representative western blotting analysis for expression of PPARα and Lipin-1. (**B**) Real-time PCR was used to determine the mRNA expression of *Lipin-1*, and *Fasn*. (**C**) Immunofluorescence staining of PPARα and Lipin-1 presented at 600× magnification. ^###^ *p* < 0.001 vs. normal group; * *p* < 0.05, *** *p* < 0.001 vs. EtOH group; ns, not significant.

Chronic alcohol consumption notably reduced the protein level of PPARα and promoted that of Lipin-1 relative to the levels in the normal group (Figure 5A). In alcohol-exposed mice, NK-mediated stimulation upregulated PPARα protein levels and decreased Lipin-1 protein levels (Figure 5A). In alcohol-exposed mice, lipogenesis-related factors, such as *Lipin-1* and *Fasn*, had markedly upregulated mRNA levels relative to those in the normal group (Figure 5B). However, NK treatment markedly reduced *Lipin-1* and *Fasn* mRNA levels. Immunofluorescence staining revealed that chronic alcohol consumption apparently decreased PPARα levels and increased Lipin-1 levels relative to those in the normal group. Moreover, NK remarkably reversed the above alterations (Figure 5C). The above results confirmed that NK could attenuate lipid accumulation to reverse ALD.

### 2.6. Nur77 May Be Involved in the Regulatory Effects of NK against the P2X7r-Mediated Inflammatory Response in ALD Mouse Livers

**Figure 6:**
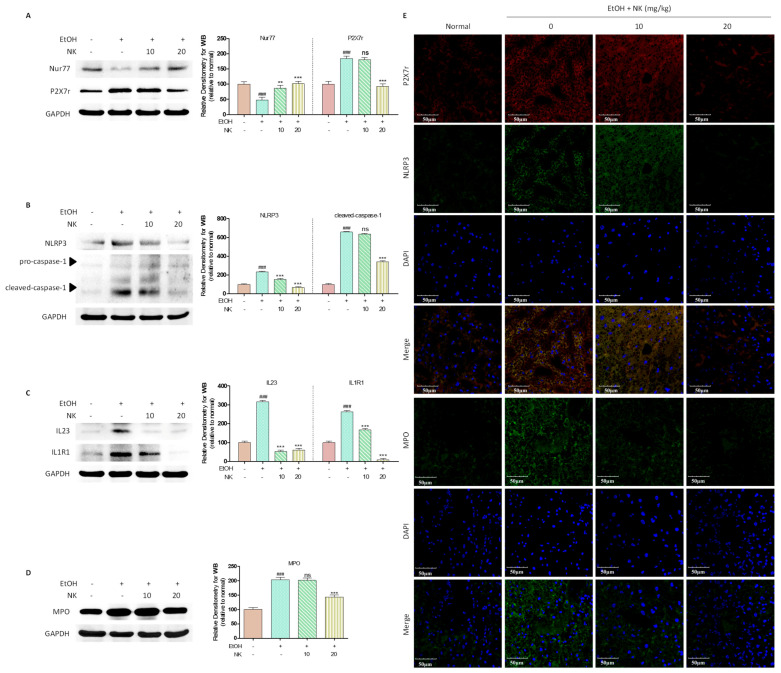
Nur77 May Be Involved in the Regulatory Effects of NK against the P2X7r-Mediated Inflammatory Response in ALD Mouse Livers. (**A**) Representative western blotting analysis for expression of Nur77 and P2X7r. (**B**) Representative western blotting analysis for expression of NLRP3 and caspase-1. (**C**) Representative western blotting analysis for expression of IL-23 and IL1R1. (**D**) Representative western blotting analysis for expression of MPO. (**E**) Immunofluorescence staining of P2X7r and NLRP3 presented at 600× magnification. Immunofluorescence staining of MPO presented at 600× magnification. ^###^ *p* < 0.001 vs. normal group; ** *p* < 0.01, *** *p* < 0.001 vs. EtOH group; ns, not significant.

Chronic alcohol consumption dramatically downregulated Nur77 protein and upregulated P2X7r protein compared to the levels in in the normal group (Figure 6A). In alcohol-exposed mice, NK treatments notably upregulated Nur77 protein and decreased P2X7r protein (Figure 6A). Enhancement of the NLRP3 inflammasome was observed in alcohol-exposed mice and was accompanied by upregulation of caspase-1, IL-23, and IL1R1 (Figure 6B,C). Even after alcohol exposure, NLRP3, caspase-1, IL-23, and IL1R1 protein levels significantly decreased after NK therapy compared to those in the alcohol group. As revealed by immunofluorescence analysis of P2X7r and NLRP3, alcohol exposure markedly upregulated P2X7r and NLRP3 expression, while NK treatment markedly reduced P2X7r and NLRP3 expression (Figure 6E). Based on the above findings, NK ameliorated ALD through the Nur77/P2X7r signaling pathway.

After chronic alcohol feeding, myeloperoxidase (MPO) expression markedly increased relative to that in the normal group (Figure 6D). MPO, a mature-macrophage marker, accumulates within activated liver macrophages. The MPO protein level dramatically decreased after NK administration, even after alcohol stimulation. Positive MPO expression was detected via immunofluorescence staining. Chronic alcohol consumption markedly upregulated MPO in mice, and NK-mediated stimulation apparently decreased MPO expression (Figure 6E).

## 3. Discussion

ALD represents a major global factor in morbidity and mortality [23]. Alcoholic fatty liver is the earliest-identified and most common form of ALD. Fatty acid accumulation within hepatocytes, arising from ethanol-induced toxic metabolic reactions and a complex immune response, constitutes a fundamental cause of ALD occurrence [24]. This disease manifests as various liver injuries, including simple steatosis, alcoholic hepatitis, and cirrhosis [25]. The present study presents findings on a molecular mechanism by which NK, a furanocoumarin glycoside initially isolated from Angelicae gigas, improves ALD. NK treatment mitigated both liver inflammation and lipid accumulation in EtOH-induced ALD models. By targeting Nur77-mediated P2X7r activation, NK alleviated inflammation and steatosis during chronic ALD, suggesting its potential as a therapeutic agent for these conditions.

Alcohol abuse induces hepatocyte steatosis through the presence of complex toxic metabolites. This, in turn, initiates immune responses and stimulates the production of chemokines and cytokines, leading to excessive recruitment of neutrophils and monocytes to the liver, ultimately resulting in severe inflammation [26]. Furthermore, chronic alcohol consumption can compromise the integrity of the gut barrier, leading to increased gut permeability and ectopic immune activation. This transfer of bacterial or microbial metabolites from the intestinal mucosa to the liver is a crucial pathological process in the progression of alcoholic steatosis to ASH, potentially sensitizing liver macrophages. In this process, macrophages can lead to direct inflammation and hepatocyte injury during ALD [27]. In the present study, NK ameliorated macrophage activation and inhibited the entrance of extracellular proinflammatory factors, which is crucial for combating ALD. A peritoneal-macrophage-conditioned medium containing LPS and ATP was used to mimic cell communication between macrophages and hepatocytes. It has been established that macrophages facilitate hepatocyte injury by releasing inflammatory substances. However, this study revealed that NK can impede the release of inflammatory components by activated macrophages, thereby leading to significant inhibition of hepatocyte injury and a reduction in inflammatory factors. Lipogenesis has been increasingly suggested to be related to ALD occurrence. Sterol regulatory element-binding protein 1 (SREBP1) is a key transcriptional regulatory factor for lipogenesis [28]. Lipin1 is a phosphatidate phosphatase enzyme that is important for liver lipid metabolism. According to the current findings, NK might suppress SREBP1 and Lipin-1, further alleviating the development of ALD. In addition, peroxisome proliferator-activated receptor α (PPARα) is an important transcriptional regulatory factor for different enzymes related to lipid metabolism. PPARα transcriptionally modulates target gene expression levels by heterodimerizing with transcription factors [29]. This study proved that NK could activate PPARα through chronic alcohol administration, thereby mitigating lipid accumulation and chronic ALD occurrence.

The toxicity of ethanol, oxidative metabolites and lipid metabolic disturbances to liver cells induces ATP production outside of cells, thus activating P2X7r on cells and participating in the inflammatory response [15]. Binding between the purinergic receptor P2X7 and ATP can activate the inflammasome, ultimately resulting in NLRP3 assembly with ASC, procaspase-1 cleavage, and caspase-1-regulated pro-IL-1β transformation into biologically active and secreted forms [30]. Cleaved caspase-1 is an effective proinflammatory factor related to various autoimmune inflammatory responses and has critical effects on the alcohol-exposure-induced inflammatory response [31]. The results of the present study provide direct evidence that NK could inhibit the P2X7r signaling pathway and strongly abrogate the inflammatory response in both hepatocytes and macrophages. Nur77 deletion led to liver inflammation, which was evidenced by inflammatory-factor generation and an enhanced inflammatory response. Chronic hepatic inflammation may induce cell injury and lipid deposition within hepatocytes [32]. In addition, Nur77 evidently enhances the transport of cholesterol from peripheral cells into the liver, reduces liver lipid accumulation and decreases intestinal lipid uptake [33]. In particular, it was observed that the Nur77 regulator suppressed acute and chronic liver inflammation in obese animals but also ameliorated nonalcoholic fatty liver disease (NAFLD) by reducing lipid synthesis while enhancing anti-inflammatory and antioxidative status [34]. Furthermore, hepatic Nur77 overexpression modulates the plasma lipid profile and reduces hepatic triglyceride levels [35]. However, the exact molecular mechanism by which Nur77 controls the inflammatory response and lipogenesis in the liver to fight ALD has not been determined. Many studies have used immortalized hepatocyte cell lines such as hepatocellular carcinoma HepG2 and AML-12 cells; these cell lines present liver-like functions including albumin synthesis and triglyceride secretion. Studies indicate that HepG2 or AML-12 cells most closely exemplify basal and insulin-stimulated glucose metabolism, whereas AML-12 cell injury most closely models lipid metabolism [36]. In the present study, NK ameliorated ASH by activating Nur77 in hepatocytes and macrophages. To elucidate the precise regulatory association between Nur77 and P2X7r, the present investigation used recombinant Nur77 (rNur77). rNur77 decreased P2X7r expression, lipid accumulation, and inflammatory-factor levels, suggesting that Nur77 activation reduced the inflammatory response and lipogenesis in hepatocytes. Csn-B is a naturally occurring agonist for Nur77. Csn-B specifically binds to the ligand-binding domain of Nur77 and stimulates Nur77-dependent transactivation activity towards target genes including Nur77 itself, which contains multiple consensus response elements allowing positive autoregulation in a Csn-B-dependent manner [37]. To clarify the specific regulatory relationship between Nur77 and P2X7r, Csn-B was used for the following experiments. NK inhibited the expression of P2X7r and further regulated the balance of lipids, while showing almost no difference compared with the Csn-B treatment, which meant that NK could function as an Nur77 agonist. A Nur77 deficiency model was created in hepatocytes with low Nur77 expression. Upon Nur77 gene silencing, P2X7r and Lipin-1 signaling levels within stimulated hepatocytes were greatly enhanced. These results indicate that NK can act synergistically with Nur77 to improve ALD. Moreover, NK can impede inflammation and ALD via the P2X7r pathway in macrophages and hepatocytes. The upregulation of Nur77 may influence this regulatory mechanism. Hence, Nur77 enhancement and P2X7r blockade are possible molecular anti-ALD mechanisms of NK.

**Figure 7:**
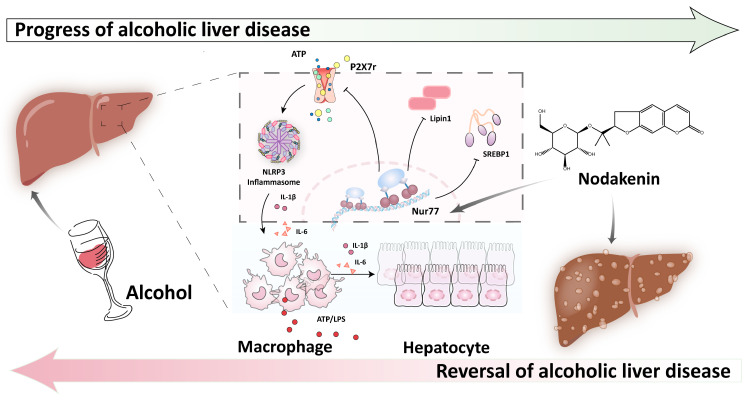
NK ameliorates hepatic fibrosis in a manner potentially involving Nur77-mediated P2X7r inflammatory signaling and lipogenesis.

In summary, our findings shed novel light on the molecular mechanisms underlying the function of the Nur77-P2X7r axis in hepatocytes during ALD. According to our results, NK mitigated ALD, which might be related to activating the Nur77-induced P2X7r-related inflammation pathway and lipid accumulation. Targeting macrophage–hepatocyte interactions during ALD may account for the beneficial outcomes of NK treatment (Figure 7). These results indicate that NK is a potential therapeutic agent for ALD. In conclusion, the prospect of NK as a clinical treatment for ALD is promising and warrants extensive exploration of the compound’s pharmacokinetics and other properties in further research. Additionally, further study should be enhanced for the translation of NK into clinical therapies.

## 4. Materials and Methods

### 4.1. Materials and Reagents

NK (purity, >98%) was acquired from Shanghai Yuanye Bio-Technology Co., Ltd. (Shanghai, China) (495–31–8) (250 mg administered). Lipopolysaccharide (LPS, 297–473–0) was acquired from Sigma-Aldrich (St. Louis, MO, USA). Antibodies against α-SMA (bs-10196R) and collagen type I (collagen-I) (bs-0578R) were obtained from Bioss (Woburn, MA, USA). Antibodies against PPARα (sc-398394), SREBP-1 (sc-365513), caspase-1 (sc-56036), IL-23 (sc-271279), ASC (sc-514414), Lipin-1 (sc-376874), Nur77 (sc-365113), NLRP3 (ab4207), P2X7r (sc-514962), and IL-1R1 (sc-393998) were obtained from Santa Cruz Biotechnology (Santa Cruz, CA, USA). Antibodies against NLRP3 (ab4207), Lipin-1 (ab181389), P2X7r (ab307718), MPO (ab208670), and GAPDH (ab8245) were obtained from Abcam (Cambridge, MA, USA). Csn-B (a Nur77 agonist, 321661–62–5) was purchased from Beyotime Biotechnology (Shanghai, CN, USA). rNur77 (ab152448) was purchased from Abcam (Cambridge, MA, USA).

### 4.2. Animals

Male C57BL/6 mice (20 ± 2 g) were provided by Changchun YiSi Experimental Animal Technology Co., Ltd. (Changchun, China) and raised within a free unit at ambient temperature with a 12 h/12 h light–dark cycle. The animals were allowed access to water and food. All the experiments complied with the requirements of the National Act on the Use of Experimental Animals (PR China). Our animal procedures were approved by the Animal Ethics Committee of Beihua University and were carried out following the Guidelines for Care and Use of Laboratory Animals of Beihua University. Mice were stochastically classified into 4 groups (*n* = 6 each): pair-fed, EtOH-fed, EtOH-fed plus NK (10 mg/kg), and EtOH-fed plus NK (20 mg/kg) groups. Mice in the EtOH-fed and EtOH-fed+NK groups were given a Lieber–DeCarli liquid diet that contained 5% (*v*/*v*) ethanol. Ethanol was administered at concentrations ranging from 0% to 4% (*v*/*v*) at 2-day intervals and later given at 5% for 28 days (Figure 4A). Animals in the pair-fed group were given a Lieber–DeCarli liquid diet that contained isocaloric maltodextrin. The NK dose was determined with reference to our prior results and literature review [21]. NK was orally administered to animals in the ethanol-fed plus NK group at a dose of 10 mL/kg body weight. Mice in the ethanol plus NK group were gavaged with NK daily for 4 weeks. NK was intragastrically administered daily during the animal experiment. Serum and liver samples were taken for further analysis.

### 4.3. Isolation of Mouse Peritoneal Macrophages (MPMs) and Mouse Primary Hepatocytes

C57BL/6 mice received peritoneal injections of fluid thioglycollate medium (Sigma-Aldrich, MO, USA) for 4 days. Subsequently, the peritoneal-cavity fluid was collected into 6-well plates containing DMEM supplemented with 10% FBS, after which the macrophages (MPMs) were isolated. Mouse primary hepatocytes were prepared both directly from excised livers following a previously published protocol and in situ through retrograde perfusion of anesthetized mice with 1 mM EGTA and blanching solution (HEPES). Following perfusion, hepatocytes exceeding 80% viability were isolated. Matrigel-coated culture plates were used for hepatocyte seeding, and the attachment medium was then replaced with serum-free medium [38].

### 4.4. Cell Culture and Treatment

AML-12 mouse hepatocyte cells (AML-12) (RRID: CVCL_0140) were obtained from iCell Bioscience, Inc. (Shanghai, China), and maintained in DMEM–F12 supplemented with fetal bovine serum (FBS; Gibco, MA, USA), insulin–transferrin–selenium (1%), dexamethasone (40 ng/mL), GlutaMAX (1%), nonessential amino acids (NEAA), and 1% penicillin/streptomycin in an incubator at 37 °C in 5% CO_2_. After exposure to 50 mM alcohol, the AML-12 cells were cultivated for 24 h with NK and cytosporone B (20 µM). MPMs were subjected to 24 h of treatment with 1 µg/mL LPS, 4 h of persistent treatment with 2 mM ATP, and a final 24 h treatment with NK. Later, the MPM supernatants were collected and used as conditioned media for 24 h culture of primary hepatocytes in 6-well plates, followed by the addition of NK (50 µM) for another 24 h of culture. HepG2 cells were exposed to 50 mM EtOH, followed by treatment with rNur77 for 1 h.

### 4.5. Cell Viability Assay

In brief, cell viability was determined using a 3-(4,5-dimethylthiazol-2-yl)-2,5-diphenyl thiazolyl bromide (MTT) assay. In brief, AML-12 cells, MPMs, and mouse primary hepatocytes were plated on 96-well plates. Following fusion, the cells were exposed to 24 h of NK treatment (0–100 µM), followed by 3 h of MTT incubation and measurement of absorbance values.

### 4.6. RT-qPCR

Total tissue and cellular RNA were extracted with a Total RNA Extraction Kit (Shanghai Promega, Shanghai, China) to prepare cDNA in accordance with the instructions of the cDNA Extraction Kit (KT211, Tiangen, Beijing, China). In line with the protocols for quantitative RT-PCR, pre-denaturation, amplification, extension, and optimization were completed using premix 2×SuperReal PreMix Plus, primers, cDNA template, 50× ROX Reference Dye, and RNase-free ddH_2_O.

### 4.7. Knockdown of Nur77 by siRNA in Murine

We seeded AML-12 cells in 12-well plates. Nur77-siRNA (5 µM) (mouse) together with the control siRNA (2 µM) (Santa Cruz, sc-37007) was added to AML-12 cells, and the silencing vector was generated with Lipofectamine^TM^ RNAiMAX transfection reagent (Invitrogen, Carlsbad, CA, USA).

### 4.8. Cell Immunofluorescence

After the cells were immobilized in 10% paraformaldehyde; permeabilized with 0.1% Triton X-100; blocked with 10% goat serum; and incubated overnight at 4C with primary antibodies of PPARα (santa cruz, sc-398394), Nur77 (santa cruz, sc-365113), IL-23 (santa cruz, sc-271279), NLRP3 (abcam, ab4207), Lipin-1 (abcam, ab181389), P2X7r (abcam, ab307718), and MPO (abcam, ab208670), the secondary antibody was added for cell incubation the next day. Later, DAPI was added to the cells, which were then observed with a fluorescence microscope.

### 4.9. Western Blotting

We utilized RIPA buffer containing a protease inhibitor for extracting cellular and liver proteins. Then, SDS-PAGE was applied to separate proteins, which were subsequently transferred to PVDF membranes (0.22/0.45 µm, GE Healthcare Bio-Science, Shanghai, China). After overnight incubation with the primary antibody, the corresponding HRP-labeled secondary antibodies were added for further incubation, with GAPDH serving as the endogenous reference. Band intensity was quantified with Quantity One Software (Quantity One V4.6.6, Hercules, CA, USA).

### 4.10. ELISA

The concentrations of murine IL-1β and IL-23 contained in the culture medium from MPMs or AML-12 cells were determined with ELISA kits (R&D Systems, Minneapolis, MN, USA) according to their specific instructions.

### 4.11. Serum Aminotransferase Assays

We utilized a serum triglyceride (TG), alanine aminotransferase (ALT), and aspartate aminotransferase (AST) assay kit (Nanjing Jiancheng Bioengineering Institute, Nanjing, China) to determine the serum ALT/AST/TG levels.

### 4.12. Histopathological Analysis

A portion of each liver tissue sample was fixed for 2 weeks in 10% neutral buffered formalin, followed by elution with alcohol and dimethylbenzene, paraffin embedding, and preparation of 5 µm sections. These sections were then subjected to hematoxylin–eosin (H&E) staining (using Chinese materials, Solarbio Technology, Beijing, China, G1120) and Oil Red O staining to assess changes in fibrous tissues. The sealed sections were examined under a phase-contrast Nikon microscope.

### 4.13. Statistical Analysis

The data represent the means from three separate assays and are presented as the means ±standard deviations. We used the GraphPad Prism program (GraphPad Prism 8.0, San Diego, CA, USA) for the calculations. *p* < 0.05 indicated statistical significance.

## Data Availability

Data are contained within the article.

## Abbreviations

ALD, alcoholic liver disease; ALT, alanine aminotransferase; AST, aspartate aminotransferase; ASH, alcoholic steatohepatitis; caspase-1, cysteinyl aspartate specific proteinase-1; IL-1β, Interleukin 1 beta; IL1R1, Interleukin 1 receptor type 1; NLRP3, NLR family pyrin domain containing 3; PPARα, Peroxisome proliferator-activated receptor α; P2X7r, purinergic ligand-gated ion channel 7 receptor; TG, triglyceride; SREBP1, sterol regulatory element-binding protein 1.
